# The Impact of Public Health Restrictions on Young Caregivers and How They Navigated a Pandemic: Baseline Interviews from a Longitudinal Study Conducted in Ontario, Canada

**DOI:** 10.3390/ijerph20146410

**Published:** 2023-07-20

**Authors:** Kristine Newman, Heather Chalmers, Arthur Ze Yu Wang, Sarah Ciotti, Luxmhina Luxmykanthan, Nicole Mansell

**Affiliations:** 1 Daphne Cockwell School of Nursing, Toronto Metropolitan University, 288 Church Street, Toronto, ON M5B 1Z5, Canada; ze.wang@torontomu.ca (A.Z.Y.W.); sciotti@torontomu.ca (S.C.); luxmhina@torontomu.ca (L.L.); mansellnicole@outlook.com (N.M.); 2 Child and Youth Studies, Brock University, 1812 Sir Isaac Brock Way, Saint Catharines, ON L2S 3A1, Canada; hchalmers@brocku.ca

**Keywords:** young caregiver, baseline interviews, pandemic, qualitative, COVID-19, longitudinal, social determinants of health

## Abstract

This qualitative research study is a part of a larger research project exploring the experiences of young caregivers aged 5–26 years and their families navigating the COVID-19 pandemic between 2020 to 2023. Data were collected from 14 young caregivers who participated in baseline interviews. The central research question guiding this study: What was, is, and will be the impact of changing public health restrictions on young caregivers and their families during the pandemic and pandemic recovery? Seven themes emerged through analysis: (1) Navigating Care During the Height of Public Health Restrictions, (2) Neighbourhood and Built Environment During the Pandemic, (3) Perceptions Towards COVID-19 and Public Health Restrictions/Efforts, (4) The Impact of Public Health Restrictions on Relationships, (5) Mental Health Challenges of Being a Young Caregiver During a Pandemic, (6) Navigating Formal Services and Supports, and (7) Recommendations from Young Caregivers. The findings from this empirical research suggest that young caregivers found it easier to navigate their caregiving responsibilities when public health restrictions and work-from-home mandates were initially implemented; however, this later changed due to challenges in finding respite from caregiving, maintaining social connections with friends, creating personal space at home, and finding adequate replacements for programs once offered in person.

## 1. Introduction

Despite the approximately 1.25 million young caregivers living in Canada [1], the majority of Canadians remain largely unaware of the lived experiences [2] of this underrepresented group of “invisible caregivers” [3]. For the purposes of this study, we defined young caregivers as a young person, under the age of 25 years old who provides care to a friend(s)/family member(s) who lives with an illness, disability, injury, and/or challenge to activities of daily living. According to the literature, young people in Canada may take on caregiving roles when someone in their immediate family becomes mentally or physically unwell due to addiction, age-related weakness, injury, or unforeseen life events [4,5,6]. For example, in Ontario (Canada’s largest province/territory), there are over half a million young people under the age of 25 years who provide physical, medical, emotional, and other forms of support to a parent, grandparent, or sibling [7,8,9,10]. International literature pertaining to young caregivers highlights their multiple responsibilities including school and paid employment in addition to caregiving [2,11,12]. The unpaid labour of young caregivers averages 14–27 h per week and represents approximately CAD 25,000 to CAD 50,000 in annual savings for the family and/or healthcare system [13]. In this way, young caregivers are subjected to a “young carer penalty” in the form of short- and long-term harms to academic, personal, social, and professional development [14]. If there are inadequate supports for young caregivers, they can experience several adverse health outcomes including stress and depression, low self-esteem, anxiety, loneliness and isolation, suicidal ideation, and difficulty with peer relationships resulting from a lack of support [13,14,15,16]. Importantly, Canada has no existing social policies to support young caregivers at the national level. Additionally, federal social welfare programs (i.e., the Caregiver Credit, the Compassionate Care Benefit, and most recently the Canada Caregiver Recovery Benefit that started in 2020 as part of relief efforts for COVID-19) are inaccessible to the majority of young caregivers, especially those with high levels of caregiving responsibilities, because the benefits rely on young caregivers earning an income and many are not of working age and/or do not have time to work due to the time they dedicate to providing care [17]. Therefore, as a group, this population can experience unrecognized and significant social, mental, physical, and economic strain.

The COVID-19 global pandemic triggered a global health crisis [18] which has significantly impacted communities across Canada and worldwide, and highlighted pre-existing inequities amongst traditionally marginalized, socially excluded, and underrepresented groups [19,20]. The unique socio-economic and socio-emotional challenges faced by young caregivers necessitates an exploration of their experiences of the COVID-19 pandemic in their own voices. A key consideration for this study is that many young caregivers and their families cared for older adults [9,21] who are more vulnerable to adverse health outcomes from COVID-19, including hospitalization and death, according to the Centers for Disease Control and Prevention and the Government of Canada [22,23]. In order to protect their loved ones, these young caregivers can face difficult choices, such as whether to return to in-person learning or engage in extra-curricular community activities that can increase the risk of harm to the older adults they are caring for. Despite anticipating social, economic, and mental health challenges/changes that individuals and families can experience, much less is known about the impact of these challenges/changes on the family unit, and no literature exists on how families are navigating these challenges, especially families with children who must provide care to a loved one who is vulnerable to COVID-19. The “sandwich generation” (people who must take care of their parents at the same time as raising their children) has gained attention among researchers and policy-makers [24,25,26] but the perspectives of their children and the contributions of a family as a unit have not been explored. Accordingly, we investigated both young caregivers and their families to provide a holistic perspective that is missing from the existing literature related to young caregivers and the sandwich generation. Furthermore, due to the novelty of the pandemic, understanding how young caregivers and their families adjust to ongoing and novel changes to policies and institutions will contribute greatly to our existing knowledge on recovery from the pandemic. Those with caregiving responsibilities have faced compounding and unique challenges including more complex care resulting from concerted public health measures aimed at reducing the community transmission of COVID-19 (with particular attention to those most vulnerable to adverse health consequences of the disease) [27], in addition to the separation from loved ones who have COVID-19 symptoms [28], and financial burdens related to caregiving that the Canada Emergency Response Benefit (CERB) does not consider [28,29]. Young caregivers and their families face additional challenges, including changes to school and social relationships, as a result of policies and fears surrounding the virus [30,31,32,33,34].

This study is part of a larger study exploring the experiences of young caregivers and their families navigating COVID-19 starting in 2020 ongoing until 2023. The data were collected from young caregivers (baseline, 6 months, 1 year, 2 years; currently conducting 6-month follow-up interviews), family members (baseline, 1 year, 2 years; baseline interviews are completed), service providers who interact with young caregivers, community leaders/allies, and policy/decision-makers at different organizations/institutions. The findings from baseline interviews with young caregivers are reported in this paper.

The purpose of this study is twofold: (1) to identify the impact of public health restrictions on young caregivers and their families; and (2) to determine the most appropriate and acceptable strategies to support families and their children who have caregiving responsibilities within and across diverse communities. The central research question guiding this study is the following: What was, is, and will be the impact of changing public health restrictions on young caregivers and their families during the pandemic and pandemic recovery?

## 2. Materials and Methods

Baseline interviews were conducted with 9 families and 3 young caregivers who joined the study without their family members. In total, 14 young caregivers joined the study. The social determinants of health [35,36] with intersectionality [37,38,39] guided the development of our interview guide alongside the lessons and findings we gleaned from a study we conducted on the experiences, challenges, and needs of young caregivers living in urban versus rural communities in Ontario, Canada [40]. Importantly, we acknowledge there is significant diversity in young caregiver populations and recognize this is not a homogeneous group.

Interviewers were hired if they had a lived experience as a young caregiver and/or had worked directly with young caregivers. Our interviewers consisted of 6 young caregivers and 1 person who worked with young caregivers in order to capture a variety of insight and lived experiences to best match participants with an interviewer who could best potentially understand their lived experiences. The person who did not have lived experience as a young caregiver had worked with young caregivers had 6 years of experience supporting young caregivers through assisting with running programs designed for them, was 27 years old, and identified as a European Canadian woman. The 6 young caregivers who were hired as interviewers were all actively young caregivers at the time data collection took place. They were aged between 23 and 27 years; 4 identified as women and 2 as men. Three of the young caregivers identified as South Asian Canadian, one European Canadian, one East Asian Canadian, and one Middle-Eastern. Four were Master’s students, one had completed an undergraduate degree, and one had completed a professional degree. Caregiving responsibilities included caring for a parent who lived with physical disability due to a car accident, a sibling living with a learning disability, a parent living with dementia, a parent living with a chronic mental illness, grandparents/parent living with disability or chronic physical and mental illnesses, and a partner living with chronic physical health challenges. Interviewers were trained in how to conduct a semi-structured interview and were provided with appropriate guidance/supervision by the investigators during interviews to ensure the data collected were both comprehensive and representative of young caregivers’ voices. This study used a longitudinal data collection approach using semi-structured interviews. Semi-structured interviews were completed at baseline with young caregivers, or young caregivers and their family members. Follow-up interviews with young caregivers will occur at 6 months, 1 year, and 2 years. Follow-up interviews with family members will occur at 1 year and 2 years. At baseline, young caregivers were asked to participate in a 30-to-45-min interview and answer questions about how the pandemic impacted their life as a whole, how their caregiving responsibilities changed (if at all), how they and their families navigated the changes to their lives caused by the pandemic/public health restrictions, and what would make life easier as a young caregiver. Findings from the baseline interviews conducted with young caregivers are reported in this article.

### 2.1. Sampling and Recruitment

Young caregivers were recruited with the help of our community partners who provide services/supports/resources to young caregivers and their families: Young Caregivers Association (served all of Ontario during the pandemic), Ontario Caregiver Organization (served all of Ontario during the pandemic), and Young Carer Program (mainly served Toronto during the pandemic).

### 2.2. Participants

Participants were required to be a young caregiver or a family member of a young caregiver. Young caregivers were given the option of participating in the study alone or with their families. The following were used as inclusion criteria for young caregivers: (1) Must be between the ages of 5 and 25 years old; (2) provide care to a family member, friend, or loved one living with illness, disability, injury, or challenge to activities of daily life; (3) live in Ontario, Canada; (4) can speak English.

There were fourteen (*n* = 14) young caregivers who participated in baseline interviews. Eleven (11) young caregivers joined with their family members and three (3) young caregivers joined the study independently. The age of young caregivers ranged from 6 to 25 years. Ten (10) of the young caregivers were between 6 and 13 years old and four were between 24 and 26 years old. The 26-year-old participant was accepted into the study since she was less than 25 years old in 2020. Ten participants (10) lived in an urban community, two (2) lived in a rural community, one (1) lived in a suburban community, and one (1) lived in a small town. Eight (8) participants identified as girls/women, four (4) identified as boys/men, and two (2) identified as non-binary. The ethnic backgrounds of young caregivers varied, with six (6) participants identifying as European Canadian, four (4) as mixed race (three (3) were South American Canadian and European Canadian; one (1) was East Asian Canadian and European Canadian), one (1) as African Canadian, one (1) as Caribbean Canadian, one (1) as East Asian Canadian, and one (1) as South Asian Canadian. The majority of participants spoke mostly English at home with three (3) young caregivers who also spoke French in equal amounts, one (1) who spoke mostly Cantonese, and one (1) who spoke mostly Tamil.

### 2.3. Data Collection and Analysis

To analyze the interviews, reflexive thematic analysis [41,42] was used by the two investigators and a team of four research assistants that included one young caregiver who was 25 years old and had been a young caregiver since the age of 8, a volunteer service provider who worked closely with young caregivers for six years with a background in early childhood education, a Ph.D. candidate in Child and Youth Studies, and a Master’s student in public health who had been engaged with research on young caregivers for 5 years. The researchers used inductive analysis, and began with a research question. Ethics clearance was obtained from Toronto Metropolitan University’s Research Ethics Board (formerly Ryerson University Research Ethics Board) on 8 July 2021 (No. 2021-216-1) and Brock University’s Research Ethics Board on 22 July 2021 (No. 21-014).

Participants were recruited through our community partners, young caregiver organizations in Canada. Once screened for eligibility (see criteria listed previously under participants subsection), participants were invited to participate in virtual interviews over Zoom which were transcribed verbatim by research assistants. The transcripts were reviewed several times by members of the research team. Those data were analyzed contemporaneously as data were collected. Initial codes were generated and initial themes emerged through data analysis. Through collaborative discussions between members of the research team, primary themes were identified. The results were written and disseminated by the research team.

## 3. Results

There were 14 young caregivers who participated in baseline interviews. Eleven young caregivers joined with their family members and three young caregivers joined the study independently. The age of young caregivers ranged from 6 to 25 years old. Ten of the young caregivers were between 6 and 13 years old and four were between 24 and 26 years old. The 26-year-old participant was accepted into the study since she was less than 25 years old in 2020.

Seven themes emerged from the interviews with young caregivers: (1) Navigating Care During the Height of Public Health Restrictions, (2) Neighbourhood and Built Environment During the Pandemic, (3) Perceptions Towards COVID-19 and Public Health Restrictions/Efforts, (4) The Impact of Public Health Restrictions on Relationships, (5) Mental Health Challenges of Being a Young Caregiver During a Pandemic, (6) Navigating Formal Services and Supports, and (7) Recommendations from Young Caregivers.

### 3.1. Navigating Care during the Height of Public Health Restrictions

Three sub-themes arose when looking at types of care. This included caregiving responsibilities, changes in the person being cared for, and protecting the family from COVID-19.

#### 3.1.1. Caregiving Responsibilities

Four participants noted that there were responsibilities they had before the pandemic that they no longer had to do during the pandemic (i.e., attending appointments). Due to appointments being on Skype or appointments not allowing more than one person in the room, many caregivers noted that they no longer had to accompany or entertain the people they cared for during these visits. One participant reported no changes in their caregiving responsibilities.

Other responsibilities ranged from feeding (1 participant), washing and general hygiene (3 caregivers), helping their grandmother up the stairs (1 participant), and walking their sibling to school (1 participant). There were also general household chores such as dishes, which one participant would do if the person being cared for was not feeling well, and laundry. Eight participants reported helping with cooking and meal preparation, ranging from making one or all the meals for the day, with one participant having to puree meals four times a day to make them easier to swallow for the person they cared for. One young caregiver (girl, 7 years old, urban, cares for older brother who lives with autism) prepared snacks for herself because her mother was busy caring for her brother. Another participant stated, “I would cook for [my grandmother], I will get her snacks, drinks and stuff like that” (boy, 13 years old, rural, cares for grandmother who is recovering from fall). Similarly, another participant would help her parents by taking care of her sister: “I’ll help make lunch for my sister a lot like, especially on like the weekend, when my mom and dad can’t do it, they’re working or something like that, I’ll make like a lot of like lunches and breakfasts” (girl, 13 years old, suburban, cares for younger sister who lives with autism). Another participant reported ordering food for the family a few times a week.

The pandemic made it more difficult to balance home and caregiving roles for two participants, with one having to move back to their home city from university and having to step out of online classes to take care of caregiving responsibilities. Additionally, two participants reported having to navigate financial support and challenges:

*“I have a lot of travel costs because of where I live, and trying to get to her, that would be the biggest thing because, like I kind of said before, I’m like doing all the organizational things, all the bills … like right now we’re kind of trying to sell the house because she’s moved and now it’s just kind of sitting there. Those kind of things end up costing me emotionally along with financially just because there’s like more stuff on my plate, but yeah, the main cost is the kind of travel and having to go back there if there is something, you just have to do in-person. And then obviously, visiting and seeing her because I want to”*.

(woman, 25 years old, rural, cares for mother who lives with early-onset dementia)

Another participant stated:

*“Definitely, financial issues have happened, in particular with us, we’ve experienced a lot of financial hardships with my dad in terms of him being not able to work anymore. That was prior to the pandemic, but the financial issues were also exacerbated because of my dad’s chronic kidney disease. He needs to use certain medical supplies, like a catheter and a condom catheter and because of that, it has resulted in additional expenses for him. And I’ve had to pay out of pocket for things like the ThickenUp that he uses every day and other types of like diapers and things like that. So I see the financial issues existed prior to the pandemic, but more so now as well and, in particular during the pandemic [in 2020]. I unfortunately lost my job due to the reconstructing of our department and, due to budgeting, that had come to an end. And I sort of was feeling the pressure as well, of not being able to financially support myself and so that did add a level of stress and burden, because of course I was a student at the time and it definitely impacted my mental health. And yeah, that was something I experienced due to the pandemic and so now I feel like I am still trying to navigate that world of being able to, you know, persist and continue to move forward”*.

(woman, 25 years old, urban, cares for father with complications from a stroke including left hemiplegia)

#### 3.1.2. Changes in Person Being Cared For

A variety of new caregiving roles arose during COVID-19. Due to less opportunity to leave the house on their own, one participant reported having to provide more company for the person they cared for: “It has almost been every day to try to engage [my grandmother living with dementia] so she doesn’t get into her hallucination state. I usually take her for a walk for an hour or an hour and a half every day. Sometimes she will sit with me at my desk while I work and she’ll do things like colouring or looking at photos, just things to make her mind try to do something”. (woman, 26 years old, urban, cares for grandmother who lives with dementia). The participant also reported the new role of consistently reinforcing/reminding her family members about the severity of COVID-19 and ensuring that their family members were adequately wearing masks in public spaces. Two participants reported having a more difficult time with their caregiving as the lockdown had caused an exacerbation of symptoms such as aggression from those receiving care. As expressed by one participant, “When my brother didn’t get help [therapy], I was really scared [as explained by mother: “Her brother gets very aggressive because he wasn’t getting any therapy as a result of lockdowns, so she was worried..um..there would be aggression in the house towards her because she was only playmate for him at the time”]” (girl, 7 years old, urban, cares for older brother who lives with autism). The other participant reported, “Like my grandmother developing dementia. That is definitely really new because we went from maybe helping her once or twice a day to now, 24/7 care. So that has felt pretty dramatic. But sometimes I wonder if maybe part of what made her dementia progress so rapidly was that she wasn’t seeing her own friends or because she was kind of isolated herself during COVID-19” (woman, 26 years old, urban, cares for grandmother who lives with dementia). Another participant reported a blend in caregiving and work, as they would have to play games with their family members during their work breaks to keep them entertained. One participant had more caregiving responsibilities because of online schooling and stated, “I had to change the way that I work during the day, because my brother was also at home doing school and sometimes I would need to watch him, so I would have to change my schedule for what I was going to do during the day” (non-binary, 13 years old, small town, cares for younger brother who lives with autism).

A majority of the participants reported the same type of care with an increased workload due to the pandemic. These included tasks such as supervision of the person(s) being cared for, which four participants reported doing; two of them having to provide 24 h supervision. As one participant described: “[My grandmother’s] dementia progressed really rapidly over the last month and before that not very often, maybe one to two times a week [she would request my help]. She was pretty independent. Now, I would say probably seven days a week because she kind of needs 24 h care or supervision. She might wander out or do something dangerous. So I would say I am constantly alert of what is going on now”. (woman, 26 years old, urban, cares for grandmother who lives with dementia)

Entertainment responsibilities ranged from once a week to everyday for some caregivers. Most participants provided comfort and company to the person they were caring for, ranging from sitting in the bathroom with them to accompanying them to appointments and general emotional support when completing daily tasks. For example, one participant reported, “Sometimes [my brother] won’t have a bath unless I’m there” (girl, 7 years old, urban, cares for older brother who lives with autism) and another participant stated, “Me and my brother share the responsibility of bringing [my mother] to appointments and bringing her anywhere she needs to be, she doesn’t drive” (woman, 24 years old, urban, cares for mother living with schizophrenia). Another participant said, “Sometimes I just watch movies with [my grandmother] or sit quite close to her while she’s watching something” (boy, 13 years old, rural, cares for grandmother who is recovering from fall). Some participants extended care in the form of emotional support to the person they cared for, one by giving hugs and the other by boosting self esteem and keeping them calm throughout the day. One participant stated, “For one of my brothers, a lot of it’s anxiety. I’m helping to keep him happy and calm”. (non-binary, 12 years old, urban, cares for mother, father, and two brothers who live different challenges including disability). Most participants had a role of entertainment, which included tasks such as playing games (i.e., Roblox, Nintendo Switch, mobile games), watching television, providing companionship on walks, or painting and reading. For instance, one participant stated they played games with their brother in between classes: “On my breaks, we would have a 30-min break and two 15-min breaks, so on my [first] 15-min break I’d play minecraft with my brother or I would have a snack with him and then on my next 15 min break, I would also play with my brother. And on my 30-min break I’d also play with my brother and sometimes I would take breaks, on my own just during the day, and I would also play with my brother” (non-binary, 13 years old, small town, cares for younger brother who lives with autism). One participant also reported that the person they cared for was physically injured during the pandemic which increased their workload, especially as it was harder to get the same support from the hospital when COVID-19 was at its peak in 2020: “We couldn’t go and see him for the first few weeks when he was there because of the restrictions that the hospital had in terms of the number of visitors that they were allowing. They didn’t really have any guests that can go in, so that definitely impacted the quality of care that we could provide to him because we weren’t allowed to go and see him. The unit that he was in had an outbreak of COVID-19 and that ultimately impacted his care once again because we weren’t allowed to go and see him” (woman, 25 years old, urban, cares for father with complications from a stroke including left hemiplegia).

There were also medical responsibilities that caregivers maintained. For instance, one participant assisted nurses during in-home visits: “We do have nurses that come into our home three times a week and they help to assist with the wound care, and so I would also assist them if, let’s say, they need a bag, or they want to discard any materials and helping them with just dressing the wound if they need assistance” (woman, 25 years old, urban, cares for father with complications from a stroke including left hemiplegia). Two participants reported administering and monitoring medication. As one participant reported, “I do have to monitor her to make sure that she’s taking her medications and then every three months she has to go and get an injection” (woman, 24 years old, urban, cares for mother living with schizophrenia). One participant also reported having to physically turn the person they cared for in the middle of the night to prevent complications such as pressure ulcers. Two participants were responsible for scheduling appointments and four participants stated that they attended these appointments with the person they cared for. One participant reported that she and her mother had to take on a supportive role for her brother living with autism that a therapist would usually provide due to the program originally available in school being cancelled due to mandated virtual schooling. This participant reported things became easier once her mother found a working solution: “Video therapy wasn’t working at all so we’ve found one therapist that will do in-person visits” (girl, 7 years old, urban, cares for older brother who lives with autism). Two participants reported having to take over responsibilities of personal support workers (PSWs), as the family was no longer comfortable having them come into the house due to the risks of contracting COVID-19. One participant expressed “I guess before the pandemic we had a PSW coming pretty frequently for bathing. And then during the pandemic we discontinued that because we weren’t comfortable with having someone moving around to different places and then coming into our home” (woman, 26 years old, urban, cares for grandmother living with dementia). The other participant stated, “I also have nurses that come into my home to help my dad with his wound dressing and even some of them don’t wear the full yellow gown and once again it’s just another reason … I don’t understand why they’re not just trying to be fully protected” (woman, 25 years old, urban, cares for father with complications from a stroke including left hemiplegia).

#### 3.1.3. Protecting My Family from COVID-19

Caregivers described numerous methods in which they ensured the safety of their families. To begin, some reported not going outside or being extra cautious when they did leave (e.g., by practicing social distancing). Some participants reported avoiding public transportation and providing rides to the person being cared for so they did not have to risk exposure on public transportation. Exposure was also avoided in other ways, such as groceries being ordered to the house, mask wearing, frequent hand washing, and receiving the vaccination when it was available. One participant captured the experience of many young caregivers in our study during the height of the pandemic:

*“And during the pandemic and even until now I’m very, very cautious about the activities that I undertake and how many people I surround myself with and just how I go about my daily life. And so, like, for example in my family, every Friday, we normally do our grocery shopping and normally I would only have like one person in my family go and do it, as opposed to all of us at once, just limit the number of people we were around. And I feel like I’ve become much more vigilant and always like wearing a mask and if I did go out and always like practicing, you know, washing my hands very frequently and always making sure to just stay as far as the distance from people as much as possible. And just like following all those extra precautions, just as anybody else would, but just being very mindful of that”*.

(woman, 25 years old, urban, cares for father with complications from a stroke including left hemiplegia)

### 3.2. Neighbourhood and Built Environment during the Pandemic

There were two sub-themes that emerged during this study. Participants varied in geographical locations, with some living in small towns, suburban communities, or rural communities, but most resided in cities.

#### 3.2.1. Benefits and Challenges of Living Outside of the City

There were two (2) young caregivers who lived in a rural community, one (1) lived in a suburban community, and one (1) lived in a small town; one of the four young caregivers moved to the rural community during the beginning of the pandemic. They shared both the challenges and benefits of living in their communities. Benefits that young caregivers living in less urbanized communities believed were positive compared to a big city included having a big yard, being less stressed due to minimized crowding in their neighbourhoods, and more personal space. As one participant described, “Being in a smaller population density and definitely at the beginning, there was a lot less stress about the cases because, like we were following where everything was and I felt a lot less stress knowing that [my mom living with dementia] was in a community that had zero cases and she wasn’t going outside of that community” (woman, 25 years old, rural, cares for mother who lives with early-onset dementia). Most participants who lived in these communities mentioned there were ample walking trails and conveniences such as groceries that could be bought locally. Alternatively, two participants lived in a community that experienced a food shortage as a result of supply issues during the height of the pandemic.

Participants who experienced food and supply shortages resulted in families having to go to nearby towns to get supplies. As one participant described, “The grocery stores got cleaned out a lot and there weren’t a lot of food options” (non-binary, 13 years old, small town, cares for younger brother who lives with autism). There were also reported challenges with both accessing and finding other resources and programs that served a rural area such as meal delivery, PSWs, and speech therapists. Additionally, some young caregivers living in small towns reported internet outages, which made attending school and completing school work difficult at times.

#### 3.2.2. Benefits and Challenges of Living in the City

The majority of young caregivers lived in a densely populated city and had lived there prior to and during the pandemic. Participants living in cities reported many benefits and challenges. Seven young caregivers reported that parks, grocery stores, and malls were conveniently located nearby. Four participants reported being able to access supplies and medications, including one that had supplies delivered to their home. Another reported benefit of living in the city related to accessibility, including access to reliable and affordable internet, to transportation via the bus, and to timely emergency care. One young caregiver reported the benefit of having a lot of neighbours, and another stated that they were still able to access nature and open spaces while living in the city.

Participants living in densely populated cities reported challenges such as exposure to individuals not adhering with masking practices, and parks being busy or crowded. As reported by one participant, “[My grandmother living with dementia] really likes to go to the park. And the park is super busy and same with all the grocery stores in Chinatown. She really likes going to those. We didn’t know what was going on at that point so we didn’t feel safe going out there because she probably wouldn’t know to distance from people or wear a mask” (woman, 26 years old, urban, cares for grandmother living with dementia). In one case, a young caregiver’s family moved out of the city into a rural community strictly due to the high number of cases: “We wouldn’t have moved [to Nova Scotia] at all if it wasn’t for the pandemic, we would have stayed in Montreal” (woman, 25 years old, rural, cares for mother who lives with early-onset dementia).

### 3.3. Perceptions towards COVID-19 and Public Health Restrictions/Efforts

There were four major areas where varying perceptions towards COVID-19 were seen; COVID-19 and Public Health Restrictions, Vaccines and Masks, Online Schooling, and Feelings Towards Re-opening.

#### 3.3.1. COVID-19 and Public Health Restrictions

Almost all participants reported having initial fear and uncertainty about COVID-19 and its restrictions. Many reported feeling less worried in 2021, when individuals started getting their vaccinations. Most reported fear about their family getting COVID-19 due to immunocompromisation, and some worried about their friends getting COVID-19 as well. Many participants were unable to go to places they occasionally or routinely visited due to public health restrictions and fear of the virus, which impacted their mental health. The person being cared for also was not able to go out as much, resulting in them being bored at home which created the new responsibility of entertaining them: “My mom typically went outside every day to the mall and she was no longer able to do that [because of lockdowns], so she was restless at home and I had to entertain her because she had a lack of friends and other social supports outside family” (woman, 24 years old, urban, cares for mother living with schizophrenia). One participant noted, “Mom and dad were stressed working online” (boy, 12 years old, urban, cares for younger sister who lives with multiple physical health complications), and another worried that COVID-19 had exacerbated symptoms of dementia of her grandmother due to the social isolation. Public health restrictions caused a lot of frustration, especially with its changes to emergency care. One participant reported that her father (the person she regularly cares for) was hospitalized at one point during the pandemic and her family was unable to visit due to an outbreak in the unit. The stress of being unable to ensure he was properly cared for along with the existence of an outbreak was mentally challenging for her and her family to cope with.

#### 3.3.2. Vaccines and Masks

Mask mandates were perceived as positive by most caregivers, but some participants expressed annoyance with it sticking to their face, making it difficult to breathe, general discomfort, and inconvenience of wearing it over extended periods of time (i.e., at school). As one participant expressed, “Well, I’ve never liked wearing masks. Sometimes I like wearing masks, like after hockey and my face is all red and sweaty and people can’t see my face or after like sports or when I’m in like a bad mood and I don’t want people looking at me, I guess the mask comes in handy then, but it’s hard to breathe in all day at school” (non-binary, 13 years old, small town, cares for younger brother who lives with autism).

With respect to vaccines, there was a general willingness from young caregivers to receive it, even when met with some disagreement from their family members. However, there was some worry and hesitation due to fear of needles, fear of its novelty, and fear of side effects, especially if the person receiving the vaccine was immunocompromised. As one participant reported, “Yeah, I feel like I was always pro-vax and it was never an issue of whether or not I was going to get the vax, I know that. I was a little bit worried about my mom getting her vaccines, because of some of the side effects, and you know, we, my brother and I, were a little bit like extra cautious when she did get [the vaccine] in case she got any symptoms, or anything like that. But luckily there weren’t any issues” (woman, 24 years old, urban, cares for mother living with schizophrenia).

#### 3.3.3. Online Schooling

Many young caregivers experienced online schooling during the pandemic. Some caregivers (mostly those in their 20s) liked the transition to online learning because they were able to learn at their own pace and were able to be home more often to look after the person they were caring for. As reported by one participant, “I think one of the silver linings and one of the blessings in disguise, that happened because of COVID-19 has been virtual [learning]. And for me, navigating school is a big one, because when I went to university all of the classes were in person. And so having everything transition to being online really opened up a huge door for me where I could be at home and help take care of my dad while also balancing school. And the same thing with like employment as well, and that has been a really huge asset” (woman, 25 years old, urban, cares for father with complications from a stroke including left hemiplegia). However, some caregivers (mostly those younger than 18) were worried about online school for reasons not limited to being afraid of technical issues such as poor internet connectivity, keeping up with assignments, and the general adjustment to the online environment. As reported by one participant, “I was worried, yes, ‘cause when I first started online school I didn’t know if I was handing in my assignments correctly or if I was getting my marks back or anything and I didn’t go on meets like this with my teacher at first” (boy, 13 years old, rural, cares for grandmother who is recovering from fall). Additionally, being home for school caused caregiving duties to overlap, and some participants (those younger than 18) had to skip online classes to supervise or assist the person they were caring for.

#### 3.3.4. Feelings towards Re-Opening

Most young caregivers were happy with the idea of COVID-19 lockdowns ending in the middle of 2021. Likewise, they reported feeling less anxious in 2021, as they were more knowledgeable and accepting of the virus and felt happy with the hope that COVID-19 was ending. Young caregivers were excited to see their teachers/friends again and happy to go back to extracurriculars that were not available during the lockdown. However, one caregiver reported frustration with the downshift in levels of concern despite the ongoing prevalence of the pandemic.

### 3.4. The Impact of Public Health Restrictions on Relationships

The lockdown caused by the pandemic had various effects on friendships and family dynamics.

#### 3.4.1. Friendships

Most participants reported not being able to see friends in person due to school closures and the fear of catching COVID-19 from friends. Some participants were able to go to outdoor spaces such as the park to see their friends. Participants reported using texting, phone calls, social media (Snapchat, TikTok, Discord, Instagram, Facebook Messenger), and applications (Zoom, FaceTime, Google Meet) to keep in touch with friends. Some caregivers reported connecting with neighbours and extended family for socialization. They also described how difficult it was to stay in touch with friends and that it was not ideal to use technology to maintain relationships.

#### 3.4.2. Family Dynamics

A majority of caregivers reported feeling closer to their immediate family after spending so much time at home with them. Spending that much time also allowed a few younger caregivers to gain more privileges within the house, such as being trusted to use the stove.

Being so close to family also had disadvantages, such as more conflicts at home and a crowded feeling. Some caregivers felt as though they received less attention from their parent(s) in the home due to the person being cared for taking up all their time. They also felt that they had to spend more time with the person being cared for, which created more stress, burnout, and conflict in the family. As one participant noted:

*“I feel like having lived in a crowded area almost made [my mental health] worse because we were trying to keep [my grandmother living with dementia] inside all the time which means usually I had to entertain her or talk to her. Also, my grandmother’s dementia is that she has pretty severe hallucinations. She can get really caught up in a fit and sometimes other family members aren’t able to de-escalate her but somehow she is responsive to me”*.

(woman, 26 years old, urban, cares for grandmother living with dementia).

A few young caregivers and/or their family members lost their jobs due to the pandemic, which contributed additional financial stress, especially for those who were responsible for paying for medical supplies. A few caregivers expressed needing more time alone. For example, “I felt like I was trying to go in my room a lot more [sort of laughing]. Like have my own space. Then again, I am getting older and stuff. But I just really liked my alone time during COVID-19” (girl, 12 years old, urban, cares for mother and two sisters who live with challenges to activities of daily living as a result of a car accident). Some participants were upset with not being able to see other family members due to the risk of catching COVID-19. Only one caregiver reported no change in their family dynamic during the pandemic because, even before the pandemic, they were home-schooled, mostly stayed at home, and their parents could not work due to living with a disability and relied on welfare/social assistance programs.

### 3.5. Mental Health Challenges of Being a Young Caregiver during a Pandemic

As a result of closures of public indoor spaces and lockdowns, many young caregivers experienced mental health issues and had utilize outdoor spaces or figure out ways to cope at home.

#### 3.5.1. Mental Health

Mental health issues were a significant area of concern during the pandemic. Most caregivers reported feeling anxious/fearful of COVID-19 as well as isolated/lonely (i.e., missing their friends), especially since they relied on friends heavily for support and for a distraction from their caregiving responsibilities. As expressed by one young caregiver:

*“I think I mentioned this earlier, but my mental health was already kind of low because of the stress of being a student and being a caregiver and so then adding in the stress to the pandemic gave me a lot of—it just worsened what I was going through, because I didn’t have those like outlets. Like I couldn’t just go out to a restaurant and have a nice night out with my friends and just shoot the shit or blow off steam in any way, so it was just like … And even when you were able to do things, it still was not like fully relaxing because you still have to be like, “What are the regulations?”, “Can I even do that thing?”. There were so many levels of steps before you got to the place and then, when you got to the place, you’re only getting like 75% of the experience. So, it just became like no way to let off stress, so just build up the stress and anxiety”*.

(woman, 25 years old, rural, cares for mother who lives with early-onset dementia)

One caregiver reported feeling fear at home because the person they cared for had become more physically aggressive without accessing their therapy. Anxiety was a prevalent feeling amongst the caregivers as well (i.e., anxiety about potentially getting sick as a caregiver and not being able to fulfil caregiving responsibilities, anxiety about adjusting to in-person work/education when the lockdown was over, anxiety about not being able to financially support the family, generalized anxiety related to the pandemic). Some caregivers reported feelings of annoyance and anger, for example, due to entertaining the person being cared for during school breaks instead of being able to have proper breaks from caregiving duties. Participants often felt exhausted and burnt out and struggled to still have time for self-care. As expressed by one participant, “There’s so many days, where I feel like exhausted or like I’m so frustrated about something going on, like maybe an assignment or something like a friendship conflict or something and I have to come home and care for her it’s like it’s like—it’s a lot” (woman, 24 years old, urban, cares for mother living with schizophrenia). One caregiver expressed that they were upset they were not able to go to the gym anymore, which had been a big factor in maintaining their mental health.

#### 3.5.2. Coping Strategies

Young caregivers described various methods of coping during the pandemic. Some turned to video games (mobile and console gaming) and social media (with websites such as Facebook, Discord, Twitter, TikTok, and LinkedIn) as a way to combat boredom, relieve stress, and stay in touch with friends. One caregiver reported joining a Facebook group for caregivers to gain support: “I guess reading about other people’s stories and feeling less alone and knowing that other people did have similar issues—that kind of helped” (woman, 26 years old, urban, cares for grandmother living with dementia). Some turned to parents and talked to friends on the phone or via video calls. Caregivers with pets also reported playing with them when they felt sad or lonely. Playing outside, walks, and exercise (i.e., running outside, kickboxing, rock climbing) was also an aid in combatting boredom and stress. A few caregivers also participated in other activities such as shopping, watching television, and writing. One caregiver reported turning to a counsellor as a coping strategy.

#### 3.5.3. Recreation

For recreation, young caregivers did a variety of activities. Some accessed programs online they were unable to before because of distance and/or scheduling (e.g., the Cadet Program). Some explored different hobbies such as drawing or gaming. Some young caregivers spent their free time playing with toys such as Legos. One individual went to an in-person sports group where they played with their mask on. Other caregivers attended piano classes and dance classes virtually.

### 3.6. Navigating Formal Services and Supports

Young caregivers shared their experiences of finding support during the pandemic to address different challenges they faced. Additionally, navigating changes in services/supports for the person being cared for as a result of public health restrictions posed a challenge for many participants.

#### 3.6.1. Supports Accessed by Young Caregivers

There were various supports accessed by young caregivers that assisted them during the pandemic. To begin with, one caregiver reported relying on Employment Insurance after losing their job and felt that it was enough to cover expenses. A number of young caregivers attended the programs specifically designed for young caregivers (i.e., Young Carer Program, Young Caregivers Association, Ontario Caregiver Organization). Some relied on the support of nurses for wound care of the person being cared for, and some relied on PSWs for help with washing, bathing, and meal preparation. For example, “My grandmother has PSWs that come every so often to help her while me and my mom are at work … PSWs wash and bathe her. But they also do all the same things I do when I’m not there to help her” (boy, 13 years old, rural, cares for grandmother who is recovering from fall). Caregivers also relied on other family members for emotional and tangible support, while some relied on friends or neighbours. Some participants relied on stuffed animals for support, and some turned to pets. A few caregivers turned to Google and internet forums such as Reddit and Facebook groups to seek advice on their caregiving roles. As one participant described, “I have used Reddit forums. It’s hard to find information specific to your situation, of course, I think I kind of have a little bit more of a unique situation. But I did find a few Reddit forums and things like that of people in similar caregiving roles and I kind of just like reading about their experiences. I never really contributed to it, but I did like see what they wrote and it kind of like made me feel a little bit like okay, like it’s not just me going through this you know” (woman, 24 years old, urban, cares for mother living with schizophrenia).

#### 3.6.2. Changes in Formal Services and Support for Person(s) Receiving Care

Participants had mixed experiences when it came to virtual appointments. Some participants felt it was easier to attend virtual appointments because of distance and saving time and energy not having to travel to in-person locations. For example, “I was already doing a lot of things from another city, so I was already doing a lot of phone calls and things like that. It did, I think, become a little bit easier to get things done on the phone or through email with the pandemic, because people got more used to like digital signatures and things like that. Previously I had to explain my situation and tell them what’s going on in order for me to sign things digitally” (woman, 25 years old, rural, cares for mother who lives with early-onset dementia). Some participants reported negative experiences with formal services being offered virtually. For example, the lack of face-to-face communication was thought to inhibit the ability to convey vital information. Young caregivers also reported it was harder to reach healthcare workers to make appointments due to a reduced number of work days, and an increased difficulty accessing healthcare services: “For example, my dad is someone that needs physiotherapy and he needs to practice exercises every day—being able to find someone that could come into our home to do it with him was really difficult. Because of course majority of things shifted to be, you know, not in person anymore, so that was a huge impact and I find just navigating access to services in general really difficult, but I do think that … because of the pandemic, I think it definitely made it even more so [difficult]” (woman, 25 years old, urban, cares for father with complications from a stroke including left hemiplegia). Additionally, there were a lot more miscommunications when scheduling with physicians. When in-person appointments did occur, participants reported not being able to go into the appointment with their family members, which increased communication errors. One participant worried that the person they cared for would not receive enough care in the hospital due to the heavy workload of healthcare workers.

There were also disruptions in other formal services. Some caregivers reported that the person they cared for was no longer able to access social support services that they had once relied on, such as church and community centres:

*“Yeah, there was this one community program that [my grandmother] could go to for one day of the week that was no longer running in-person. The program was just one, single day of the week, but it felt really different because usually when she [was] away for that day, everyone gets to relax for the day. But yeah, we didn’t have that during COVID-19 … they started doing half an hour zoom sessions with the people that would go to the program. After a while, my grandmother wasn’t super engaged in it anymore, she would talk or do other things on her own during the program. We still had to watch her very closely when she was doing that. Sometimes I think because they were trying to cater to a broader audience—usually the day program is in Chinese so she can understand what is going on, but this was frequently in English so maybe that’s why she wasn’t as excited about it”*.

(woman, 26 years old, urban, cares for grandmother living with dementia)

Another caregiver reported being inconvenienced by the changes as they had to postpone moving the her mother (person being cared for) into an Assisted Living Facility due to the pandemic:

*“The main thing that happened is that, right before the pandemic, I was talking to my mom and we were like in the process of exploring her moving into an Assisted Living facility and I pretty much just gotten her to the point where she was like starting to be willing to like actually consider it and then the pandemic happened, and they all shut down. And they obviously weren’t taking people in, so we couldn’t do tours, so it all got put like just on the back burner, and it was like over a year until I felt like I could bring it up again. And then when I did, she didn’t feel like she wanted to do it anymore. And so, I was like even further back from step one so that was really, really, really frustrating and difficult”*.

(woman, 25 years old, rural, cares for mother who lives with early-onset dementia)

### 3.7. Recommendations from Young Caregivers and What Would Make Life Easier

When caregivers were asked about recommendations on what would make their lives easier, various ideas were shared. One caregiver stated that there should be a bigger support network for young caregivers but was unsure of what it might look like. Another suggestion involved more support programs for caregivers dealing with individuals who had mental health issues rather than just physical. There were also a few caregivers who reported the need for more respite care supports and breaks from caregiving. A “one-stop shop” for information and organizational resources was proposed, after one caregiver said that it was difficult to know all the resources that were available:

*“I wish there were more financial resources for young caregivers, and I feel like finding any type of financial help has been so cumbersome and really complicated in terms of different pages and websites and there’s just so much information and organizations out there. Just honestly, I wish there was just a one-stop-shop—have one place, that I could go to just see what was available for young caregivers and having that information be accessible, I think can be really useful. Like you know what would be … like specific home care support, for example, or having you know that resource that would be easy to access”*.

(woman, 25 years old, urban, cares for father with complications from a stroke including left hemiplegia)

One young caregiver also suggested attention be paid to the “secondary caregiver role” which involved supporting other caregivers in the family who were having difficulty coping with their caregiving responsibility:

*“I guess that one other thing that was kind of weird that I haven’t heard from other people was that I almost was like a secondary caregiver because my mom was so stressed with what was going on and the relationship with my grandmother. [the person we cared for]. Sometimes it was like looking after my mom, too. Or I guess a lot of the times yeah … I did feel like the secondary caregiver role that I felt like I had to take [on] is not necessarily something that I see in the media or hear from other people a lot. I guess looking after a very, very stressed caregiver and how to navigate that too”*.

(woman, 26 years old, urban, cares for grandmother living with dementia)

Young caregivers also recommended social engagement services that are more culturally sensitive and offered in multiple languages. Many participants wanted more financial resources for young caregivers, such as scholarships. Younger caregivers recommended having access to activities, toys, and/or extracurriculars to assist in occupying their time. Additionally, animal therapy was recommended to be an assist in coping. One caregiver expressed that online schooling had been a big barrier and that an alternative solution needed to be in place for those with internet issues and a 24/7 caregiving role. Finally, therapy and life coaches specifically designed for young caregivers were suggested.

## 4. Discussion

The aims of this study were to identify the impact of public health restrictions on young caregivers and how they navigated the pandemic, as well as to determine the most appropriate and acceptable strategies to support families and their children who have caregiving responsibilities within and across diverse communities. Through analysis, demographic factors (i.e., young caregiver age, geographic and social backgrounds) emerged as significant in evaluating the impact of the pandemic on young caregivers. As we previously noted, the majority of participants (*n* = 10) were between the ages of 6–13 years, while the remaining (*n* = 4) participants were between 24–25 years of age. Generally speaking, elementary-school-aged children and young people emerging into adulthood will face significant differences in terms of resiliency and vulnerability. Additionally, a paramount difference in legal status and social and emotional development is evident. An intersectional framework [37,38,39] rooted in the social determinants of health [35,36] emphasize the importance of exploring relevant power relations and the marginalization of identities for young caregivers.

Overall, the findings from this study suggest the impact of COVID-19 and public health restrictions on young caregivers was varied and differed based on a variety of circumstances. Differences in demographic factors such as age, family income, awareness of caregiving experience, and the needs of the person being cared for account for significant variation in the lived experiences of participants. For example, caregiving responsibilities were reported to be unchanged (i.e., being homeschooled prior to the pandemic meant there was not a big disruption during the pandemic), decreased (i.e., due to less travel to appointments with pandemic lockdowns), and increased (i.e., household chores, meal preparation, mixing of school and caregiving time). This aligns with previous research highlighting the differences in lived experiences of young caregivers in the Canadian context as a non-homogenous group [13,15,21,40,43,44,45].

Young caregivers reported initial fear and uncertainty stemming from COVID-19 and subsequent public health responses that were aimed at stopping the community spread. Pandemic lockdowns and restrictions exacerbated family stress, which was detrimental for some young caregivers, as evidenced by the concern of contracting COVID-19 and the described steps taken to minimize the risk to themselves and their family members (some with compromised immune systems). Further, the young caregivers participating in this study expressed financial concerns and/or strain due to job and income losses. Additionally, support for and attention paid to caregivers was reduced as family resources were allocated to the person needing care. More broadly, the pandemic has drawn attention to pre-existing income disparity and structural inequalities for Canadians with low incomes. Two years in, in 2022, Canada, like many parts of the world, continues to see rising inflation nationally, thus contributing to rising costs of food, gasoline, and housing, and thus placing significant strain on families who are already struggling financially, and in particular those from lower socio-economic backgrounds. This coincides with previous research findings exploring the impact of the COVID-19 pandemic lockdowns and restrictions on the mental health of young people more broadly [46,47,48,49,50,51].

Previous research findings highlight regional variations in social and health services [52,53]. This is supportive of the findings of this study, which suggest that community size and location are relevant in understanding the impact of the pandemic on young caregivers. For example, population size, access to critical infrastructure (i.e., groceries, reliable internet access, community resources/programs) were significant barriers for participants residing in rural communities. That said, those living in smaller communities pointed to the benefits of greater access to outdoor space and decreased congestion, which arguably reduced their risk contracting COVID-19 from community spread. Notably, health care, education, and social assistance are provincial/territorial jurisdiction in Canada, which contributes to the significant regional variations across the country. Commonly, major city centres have greater resources and a larger pool of professionals available within these communities; however, they also have larger populations to serve, therefore increasing wait times and community competition for access.

To mitigate vulnerability, it is critical that young caregivers are consulted and receive meaningful and relevant support in their communities. To this point, building resiliency for young caregivers involves securing and maintaining physical, social, and emotional support and resources in their homes and communities. Strong relationships with family members and friends proved beneficial for young caregivers who reported increased mental health issues stemming from isolation and loneliness and reduced coping outlets as a result of the pandemic. Young caregivers reported a loss of formal support during the pandemic as a result of pandemic restrictions.

### 4.1. Strengths and Limitations

We believe engagement and collaboration with young caregivers is a significant strength in our research and in all research with young people. Our results emphasize the experiences of young caregivers in their own voices. Given that young people have been underrepresented in research, we believe it is essential that researchers evaluate their experiences from their unique perspectives and in their own voices. Traditionally, research has focused on children’s health and children’s experiences framed within an adult-centric lens from the position of adult caregivers, so engaging with young people themselves in addition to their adult caregivers offers a unique vantage point from which to view their experiences. Additionally, to further increase representation of the voices of young caregivers, we hired many interviewers for young caregivers and only hired those with lived experience as a young caregiver or who were working/had worked directly with young caregivers. Screening questions were also used to match participants with the interviewer on our team that best fit with their experiences and socio-demographic background. For example, three (3) young caregivers of different ages provided support for their mother who lived with chronic health challenges as a result of a car accident was matched with a young caregiver on our team who, from a very young age, provided care for her mother (who also lived with challenges to activities of daily living caused by a car accident).

The limitations of this study include the small sample size. We recognize that this sample represents a small population of young caregivers from across Canada and therefore we in no way suggest that the results are generalizable. The findings from this study point to the differences in young caregiver experiences in Canada, thus reinforcing that young caregivers are not a homogenous group with apparent universal experiences. It would be beneficial to conduct future comparative studies exploring how social factors, such as, gender, cultural, regional, and socio-economic differences amongst young caregiver populations in Canada to gain a deeper understanding of how these factors influence young caregiver experiences.

### 4.2. Recommendations

We recommend that young caregivers are not only considered but consulted whenever governments and social or community-based organizations make investments in services, supports, and resources for young caregivers. It is critical that the voices of these young people are captured in order to ensure that these services meet their unique needs. The results of this study highlight how young caregivers are capable of offering recommendations and suggestions on how to improve social conditions for themselves and their families. Primarily, our participants suggest an increased investment in community-based resources that assist caregivers in maintaining their financial, emotional, mental, and physical health. Importantly, these resources should be available equitably to all young caregivers regardless of their social backgrounds (i.e., language, socio-economic and family status, culture/ethnicity, education) and/or the communities in which they reside (i.e., rural or urban). We recommend further studies that explore resiliency and vulnerability in relation to accessibility of community resources for young caregivers and their families.

## 5. Conclusions

This study presents observations focused on the experiences of young caregivers in Canada during the pandemic. The primary conclusion drawn from this research illustrates the variable impacts of the pandemic on young caregivers across the country, thus pointing to the diversity within this population, and challenging constructions of young caregivers (and young people more broadly) as a homogenous group. Young caregiver age, along with geographic and social locations, were significant factors in evaluating their resilience and vulnerability as an outcome of the pandemic. This work contributes to current knowledge on young caregivers and highlights their experiences in their own voices. As a result, this study offers a unique representation of a population that has been underrepresented in research (and social policy) to date. In order to further understand issues related to the health and well-being of young people, it is critical that researchers continue to engage young people as active participants in on-going research. We recommend future research that is focused on the longer-term impacts of the pandemic on young caregivers, including research that evaluates the experiences of young caregivers with and without community support and resources as a point of comparison. This would advance an understanding of the effects of these resources on young caregivers’ resilience and vulnerability post-pandemic.

## Data Availability

Data sufficient for the reader to validate the article findings can be made available as appropriate upon request to the corresponding author.
